# Irrigation Depth Modulates Root Water Uptake in Subtropical Citrus Orchards: Insights from Stable Isotopes and MixSIAR Modelling

**DOI:** 10.3390/plants15040537

**Published:** 2026-02-09

**Authors:** Zhenjing Tan, Min Li, You Hu, Jinjin Zhu, Yao Peng, Sheng Deng, Zichen Jia

**Affiliations:** 1 Rural Water Conservancy Research Institute, Jiangxi Academy of Water Science and Engineering, Nanchang 330029, China; 2 College of Water Resources and Architectural Engineering, Northwest Agriculture and Forestry University, Yangling 712100, China; 3 Jiangxi Key Laboratory of Flood and Drought Disaster Defense, Nanchang 330029, China; 4 Jiangxi Provincial Technology Innovation Center for Ecological Water Engineering in Poyang Lake Basin, Nanchang 330029, China

**Keywords:** citrus orchard, irrigation depth, MixSIAR model, red-soil hilly regions, root water uptake strategies, stable isotopes

## Abstract

Irrigation depth plays a critical role in regulating soil water availability and root water uptake in perennial orchards, yet its mechanistic effects remain poorly understood in subtropical red-soil hilly regions characterized by strong evaporative demand and shallow effective soil water storage. Here, a field experiment was conducted in a citrus orchard with three irrigation depths—shallow (25 cm), intermediate (50 cm), and deep (100 cm)—under a uniform irrigation amount. Soil water dynamics, root traits, and root water uptake sources across a 0–200 cm soil profile were investigated using soil moisture monitoring, root morphological analysis, dual stable isotopes (δ^2^H and δ^18^O), and the MixSIAR Bayesian mixing model. Irrigation depth markedly restructured vertical soil moisture patterns, with the 40–120 cm layer identified as the most responsive zone. Intermediate irrigation maintained the highest and most stable soil water content in this layer, whereas shallow irrigation intensified surface drying and deep irrigation failed to improve water availability within the hydraulically active root zone. Root surface area and dry mass were maximized under intermediate irrigation, indicating enhanced root–soil coupling. Isotopic analysis revealed the strongest evaporative fractionation under shallow irrigation, while intermediate irrigation substantially alleviated surface evaporation. MixSIAR results further showed that shallow irrigation progressively increased reliance on surface soil water (up to 93% in November), whereas intermediate irrigation promoted coordinated uptake from shallow, middle, and deep soil layers, with deep soil water contributing up to 30.7% in November. These results demonstrate that irrigation depth exerts a stronger control over root water uptake strategies by stabilizing water availability within the active root zone and reducing non-productive evaporative losses. Optimizing subsurface irrigation depth therefore represents an effective pathway to improve water-use efficiency in citrus orchards of subtropical hilly regions.

## 1. Introduction

Water scarcity and inefficient irrigation management are increasingly constraining the sustainable development of citrus production in subtropical regions worldwide. In southern China, citrus orchards are predominantly distributed across red-soil hilly landscapes, which are characterized by shallow soil profiles, low water-holding capacity, steep slopes, and highly uneven intra-annual precipitation patterns [1,2]. These unfavorable soil–hydrological conditions, when coupled with high evaporative demand during the growing season, frequently result in unstable water supply within the root zone. Consequently, irrigation becomes the dominant regulator of soil–plant water relations in such orchard systems [3,4]. Under the intensifying pressures of climate change, improving water use efficiency while maintaining yield has emerged as a critical challenge for irrigation management in citrus orchards located in red-soil hilly regions.

Irrigation depth is widely recognized as a key factor governing the vertical distribution of soil water and its accessibility to plant roots [5,6]. Shallow irrigation can rapidly increase surface soil moisture but often exacerbates evaporative losses, whereas deep irrigation is conducive to the formation of a relatively stable deep soil water reservoir but may reduce water availability to the most active root zones [7,8]. Previous studies have demonstrated that irrigation depth can substantially alter soil moisture stratification, root growth characteristics, and crop performance in orchard systems [9,10]. However, the regulatory effects of deep irrigation are highly contingent upon soil hydraulic properties, root system architecture, and atmospheric evaporative demand. In red-soil hilly regions characterized by rapid drainage and intense evaporation, the underlying mechanisms by which irrigation depth modulates soil–plant water relations remain insufficiently and systematically understood.

Root system plasticity plays a central role in plant adaptation to heterogeneous soil water environments. Numerous studies have shown that root length density, root surface area, and biomass allocation can dynamically adjust in response to soil moisture conditions and irrigation practices, thereby regulating water uptake efficiency [11,12]. In citrus orchard ecosystems, roots are typically concentrated in the shallow soil layers, yet their actual contribution to water absorption can vary substantially under different irrigation regimes and seasonal stages [13,14]. Reliance solely on the spatial distribution of roots is insufficient to accurately reflect the true sources of plant water, especially when significant gradients in soil moisture and isotopic composition exist along the soil profile.

Stable hydrogen and oxygen isotopes (δ^2^H and δ^18^O) provide a powerful tool for elucidating plant water sources and root water uptake strategies. The dual-isotope approach enables discrimination among precipitation, soil water at different depths, and xylem water, thereby directly revealing plant water use pathways [15,16,17]. In irrigated orchard systems, isotope-based studies have shown that different irrigation regimes can substantially influence soil water evaporation intensity, isotopic fractionation patterns, and root water uptake depth [18,19]. However, most existing research has focused on irrigation amount or frequency, with limited attention to the role of irrigation depth in regulating soil water isotopic characteristics and root water uptake dynamics, particularly in subtropical red-soil regions, where significant knowledge gaps remain.

In recent years, the development of Bayesian mixing models such as MixSIAR has provided a more robust approach for quantitatively disentangling plant water sources, allowing uncertainties to be explicitly accounted for on the basis of isotopic analysis [20,21,22]. Integrating stable isotope tracing with observations of soil moisture and root traits facilitates a systematic understanding of how irrigation depth regulates the soil–root–water continuum and influences seasonal shifts in root water uptake strategies [23,24,25]. Nevertheless, studies simultaneously combining soil moisture dynamics, root architectural responses, isotopic fractionation processes, and quantitative assessments of water sources remain limited in perennial orchard ecosystems.

Based on the above background, this study focused on a citrus orchard in the red-soil hilly region of southern China to systematically investigate the integrated effects of different irrigation depths on soil moisture distribution, root system traits, and root water uptake sources. The specific objectives were: (1) to quantify the temporal dynamics and vertical distribution patterns of soil water content under shallow, intermediate, and deep irrigation regimes; (2) to assess the effects of irrigation depth on citrus root architecture and its vertical distribution; (3) to reveal soil water evaporative fractionation patterns and their responses to irrigation depth based on δ^2^H–δ^18^O dual-isotope analysis; and (4) to quantitatively determine the seasonal contributions of different soil layers to citrus root water uptake using the MixSIAR model. By integrating hydrological, root, and isotopic evidence, this study aims to provide a theoretical basis and practical guidance for optimizing water-efficient irrigation depth in citrus orchards of red-soil hilly regions.

## 2. Materials and Methods

### 2.1. Study Area

The field experiment was conducted in 2024 in a standardized Nanfeng tangerine orchard located in Chating Village, Baishe Town, Nanfeng County, Jiangxi Province, China (27°05′N, 116°27′E). The region has a subtropical monsoon climate, with a mean annual temperature of 18.5 °C, an average annual precipitation of approximately 1845.5 mm, and a frost-free period of about 285 days. Rainfall is highly seasonal, with frequent summer drought events despite high annual precipitation totals.

The orchard consisted of 15-year-old citrus trees planted at a spacing of 4 m × 3 m, with uniform canopy structure and standard local management practices. The soil is classified as a typical red soil, characterized by strong weathering, acidic conditions, and limited water-holding capacity. Key soil physicochemical and hydraulic properties at different depths are summarized in Table 1.

### 2.2. Experimental Design

**Figure 1:**
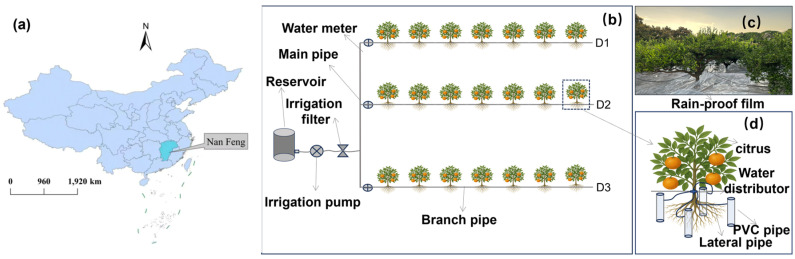
Diagrams of the deep irrigation system and distribution map of Nanfeng tangerine root systems. (**a**) Location of the study area; (**b**) The layout of the irrigation systems for different treatments in the experimental area; (**c**) A photo of laying rain shelter film on-site; (**d**) Schematic diagram of the subsurface emitter.

A single-factor field experiment was established to examine the effects of irrigation depth on soil water dynamics and citrus root water uptake. All treatments received the same total irrigation amount, equivalent to 75% of full irrigation, where full irrigation maintains the soil moisture upper limit at 100% of field capacity, while irrigation depth varied among treatments. Three irrigation depths were applied: shallow irrigation (D1, 25 cm), intermediate irrigation (D2, 50 cm), and deep irrigation (D3, 100 cm). Each treatment was replicated in separate plots within the orchard (Figure 1). In this study, three trees located in the center of each plot were selected for each treatment as three biological replicates. Considering the terraced planting pattern of the experimental site (terrace width of 4 m) and the tree spacing of 6 m × 6 m, the area of each individual plot was calculated to be 144 m^2^. All treatments were arranged using a completely randomized design.

To exclude the influence of natural precipitation during the experimental period, all plots were covered with rain proof. In this study, a 12-mil-thick polyethylene anti-aging rain-shelter film was used. The film had a light transmittance of ≥85% and was coated with an anti-fogging layer to minimize alterations in the light environment under the shelter, thereby reducing potential impacts on citrus photosynthesis. Irrigation water was delivered through subsurface emitters installed at the designated depths, ensuring precise control of water input location while maintaining consistent irrigation volume across treatments. The irrigation frequency was once every half month (15 days). Prior to each irrigation event, soil samples were collected from different soil layers in each plot (consistent with the monitored soil depths in the experiment) using a soil auger. Gravimetric soil water content was determined by the oven-drying method. An upper irrigation threshold of 75% of field capacity was applied, and the irrigation amount was calculated as the difference between the current soil water content and this threshold. A pressure-compensated drip irrigation system was used, with emitters delivering a flow rate of 8 L h^−1^. Each plot was equipped with a water meter with an accuracy of 0.001 m^3^ for real-time measurement to ensure precise irrigation application. The duration of each irrigation event was dynamically adjusted according to the required replenishment volume, emitter flow rate, and the number of trees per plot. The actual irrigation amount for each treatment was determined based on water meter readings and subsequently converted to irrigation water applied per tree (L tree^−1^).

### 2.3. Sample Collection and Analysis

#### 2.3.1. Soil Water Content

Soil samples were collected on representative sunny days in July, August, September, and November 2024, at monthly intervals. Using a soil auger, samples were taken at 20 cm intervals along the 0–200 cm soil profile. The samples were immediately placed in pre-labeled aluminum boxes and sealed. The sealed samples were oven-dried at 105 °C for 10 h until a constant weight was reached to determine gravimetric soil water content, which was then multiplied by soil bulk density to calculate volumetric soil water content (θ). For isotopic analysis, soil samples were placed in 100 mL wide-mouth plastic bottles and sealed with parafilm. The isotopic samples were stored at –20 °C to prevent degradation and ensure the accuracy of isotopic information. Volumetric soil water content was calculated using the following equation:

(1) $$\theta =\theta_{m}\times \rho$$

In the equation, θ represents volumetric soil water content (m^3^ m^−3^); θₘ is the gravimetric soil water content; and *ρ* is the soil bulk density (g cm^−3^).

#### 2.3.2. Stable Isotope Sampling and Measurement

Soil samples for isotopic analysis were collected simultaneously with soil moisture sampling and immediately sealed in airtight containers to prevent evaporation. Samples were stored at −20 °C prior to water extraction. Soil water was extracted using a low-temperature cryogenic vacuum extraction (CVE) system. Extraction efficiency was calculated as:

(2) $$\alpha =\frac{n_{2}-n_{1}}{m_{2}-m_{1}}$$

where α is the extraction efficiency (%); $n_{2}$ and $n_{1}$ are the weights of the collection tube after and before extraction (g), respectively; and $m_{2}$ and $m_{1}$ are the weights of the sample tube before extraction and after oven-drying (g), respectively. Extraction efficiency was maintained between 98–102% to ensure the validity of isotopic information [26].

Stable isotope compositions of soil water (δ^2^H and δ^18^O) were measured using a liquid water isotope analyzer (LWIA-45EP, Los Gatos Research, Mountain View, CA, USA). Citrus xylem water was extracted from one-year-old shoots collected at midday, with phloem carefully removed prior to extraction. To minimize potential spectral interference from organic compounds, xylem water isotope analysis was conducted using an isotope ratio mass spectrometer (IRMS, Iso-prime Limited/Elementar UK Ltd., Cheadle Hulme, Greater Manchester, UK). A correction of 8.1‰ was applied to δ^2^H values to account for systematic depletion associated with cryogenic extraction.

All isotope ratios are reported relative to Vienna Standard Mean Ocean Water (VSMOW) as:

(3) $$\delta =\frac{R_{Sample}-R_{Stand}}{R_{stand}}\times 1000$$

where δ is the isotopic composition (‰), $R_{Sample}$ is the isotope ratio of the sample (‰), and $R_{Stand}$ is the isotope ratio of the reference standard VSMOW (‰).

Citrus plant samples were collected at noon from one-year-old shoots, with phloem carefully removed to retain only xylem, sealed, and refrigerated prior to vacuum extraction. To account for potential δ^2^H depletion during extraction, an 8.1‰ correction was applied [27]. Atmospheric precipitation samples were obtained using an evaporation-minimizing rainfall collector, with single-event samples refrigerated to prevent evaporative fractionation.

#### 2.3.3. Root Sampling and Analysis

Root samples were collected in October during the fruit maturity stage. Root samples were collected using a soil auger, with a sampling volume of 954.23 cm^3^ for each soil layer. After sampling, root materials were carefully washed to remove adhering soil and impurities, oven-dried to a constant weight, and weighed to determine dry mass. The reported fine root dry weight (RDW) was standardized to the sampled soil volume, ensuring comparability of data among treatments and across different soil layers. Sampling was conducted at a horizontal distance of 1 m from the tree trunk and extended vertically to a depth of 200 cm at 20 cm intervals. Roots were carefully washed, and non-citrus roots were removed manually. Cleaned roots were stored at −20 °C prior to analysis.

Root samples were scanned at 300 dpi using an Epson V700 scanner (Seiko Epson Corporation, Suwa, Nagano, Japan). Root length density (RLD) and root surface area (RSA) were quantified using image analysis software. Subsequently, roots were oven-dried at 70 °C for at least 72 h to constant weight for determination of root dry weight (RDW).

### 2.4. Statistical Analysis and Isotope Mixing Modelling

All experimental data were processed using Microsoft Excel 2021. Statistical analyses were conducted using SPSS 27.0. One-way analysis of variance (ANOVA) was applied to evaluate differences among irrigation treatments, and Duncan’s multiple range test was used for post hoc comparisons at a significance level of *p* < 0.05. Figures were produced using Origin 2021.

The Bayesian mixing model MixSIAR was applied to quantify the proportional contributions of soil water from different depth intervals to citrus root water uptake. The model was implemented in R (version 4.2.2) using uninformative (uniform) priors. Both residual and process error structures were enabled to account for isotopic variability and source heterogeneity. Markov chain Monte Carlo (MCMC) simulations were run with three chains, each with 100,000 iterations, a burn-in period of 50,000 iterations, and a thinning interval of 50. Model convergence was evaluated using Gelman–Rubin diagnostics (values < 1.05) and visual inspection of trace plots, with the results showing that the Gelman–Rubin diagnostic values of all parameters were less than 1.05 and the trace plots exhibited stable trends without significant fluctuations, thus confirming a good model convergence performance. Results are reported as posterior means with 95% credible intervals.

Based on soil water isotopic dynamics and hydrological processes, soil water sources were classified into three depth intervals: shallow (0–40 cm), intermediate (40–120 cm), and deep (120–200 cm). The 0–40 cm layer was the primary root and water uptake zone, showing peak root traits and the highest contribution across treatments, with soil moisture strongly influenced by evaporation and irrigation. The 40–120 cm layer functioned as a relatively stable active root zone and represented the key responsive layer to irrigation-depth regulation, whereas the 120–200 cm layer mainly served as a deep supplementary water source during drought periods. This classification ensured isotopic separability among sources and reflected functional differences in root water accessibility.

Following the widely accepted assumption that no isotopic fractionation occurs during root water uptake and xylem transport, citrus xylem water isotopic composition was assumed to directly reflect source water signatures.

## 3. Results

### 3.1. Temporal Dynamics of Soil Water Content (SWC) Across Different Soil Layers

**Figure 2:**
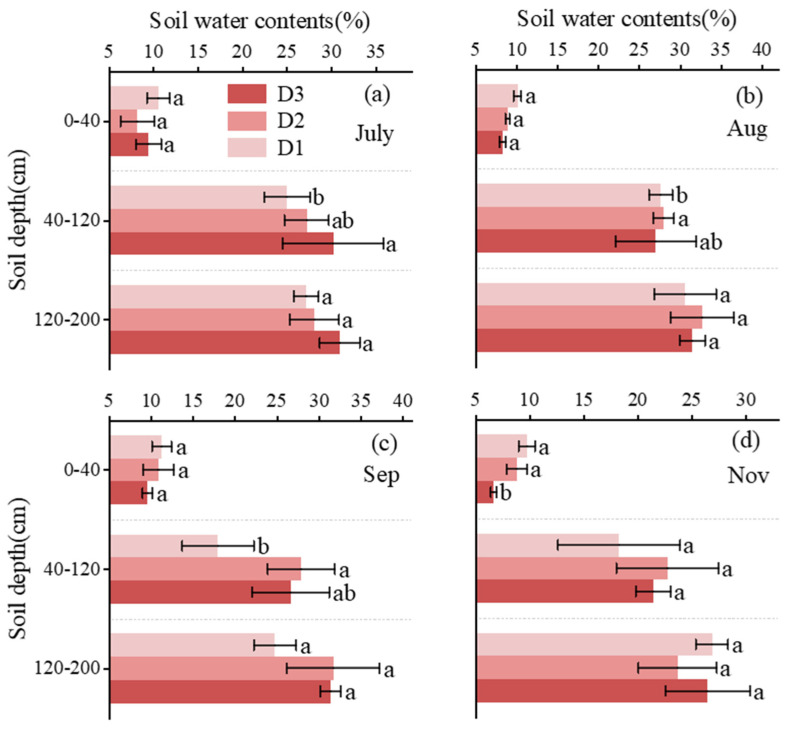
(**a**–**d**) Monthly variations in soil water content (SWC) at different soil depths (D1, shallow; D2, intermediate; D3, deep). Vertical bars indicate standard deviations. Different lowercase letters indicate significant differences among irrigation treatments (D1, D2, and D3) within the same soil layer (*p* < 0.05).

Soil volumetric water content (SWC) exhibited pronounced temporal and vertical variability under different irrigation depth treatments (Figure 2). Across all sampling months and treatments, SWC generally increased with soil depth, with the lowest values consistently observed in the 0–40 cm layer, indicating a strong susceptibility of surface soil to drying.

Across the growing season, soil water content (SWC) exhibited distinct treatment- and depth-dependent patterns. In July, SWC in the 40–120 cm hydraulically active zone was highest under deep irrigation (D3, 30.24%), significantly exceeding that under shallow irrigation (D1, 25.07%; *p* < 0.05) and comparable to moderate irrigation (D2, 27.28%), while no significant differences were observed among treatments in the 0–40 cm or 120–200 cm layers. In August, moderate irrigation (D2) resulted in the highest SWC in this layer (28.04%), which was slightly but significantly greater than D1 (27.69%; *p* < 0.05), whereas D3 (27.10%) did not differ significantly from either treatment. This pattern persisted in September, with D2 maintaining the highest SWC (27.94%), representing an increase of more than 50% relative to D1 (*p* < 0.05) and remaining comparable to D3 (26.66%). By November, SWC in the 0–40 cm layer was lowest under D3 (6.72%), significantly lower than under D1 (9.78%) and D2 (8.87%), whereas no significant treatment effects were detected in deeper soil layers.

Although the rain-shelter film used in this study was designed with high light transmittance and enhanced ventilation to minimize microclimatic interference, it may still have slightly reduced surface soil evaporation compared with open-field conditions, potentially resulting in lower soil water content in the 0–40 cm layer. However, all treatments were subjected to the same sheltered conditions, and the effects of reduced evaporation were therefore consistent across treatments. Consequently, such evaporation differences did not alter the irrigation-depth–driven vertical distribution of soil moisture, and the water regulation effects within the active root zone (40–120 cm) remain robust and reliable.

### 3.2. Response Traits and Vertical Distribution Patterns of Citrus Roots

**Figure 3:**
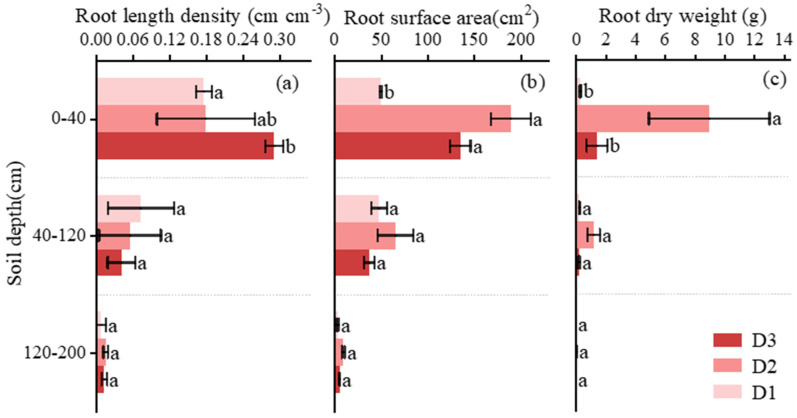
(**a**–**c**) Root length density (RLD), root surface area (RSA), and root dry weight (RDW) of citrus across the soil profile under different treatments. Different lowercase letters indicate significant differences among irrigation treatments (D1, shallow; D2, intermediate; and D3, deep) within the same soil layer (*p* < 0.05).

Root length density (RLD), root surface area (RSA), and root dry weight (RDW) exhibited similar vertical distribution patterns across irrigation treatments, with all three indices peaking in the 0–40 cm layer and declining sharply with increasing soil depth (Figure 3).

In the 0–40 cm layer, RLD under deep irrigation (D3, 0.29 cm cm^−3^) was significantly higher than that under shallow irrigation (D1, 0.17 cm cm^−3^; *p* < 0.05), while no significant difference was observed between D3 and D2 (0.18 cm cm^−3^). Across deeper soil layers, RLD did not differ significantly among treatments.

Root surface area consistently reached the highest values under intermediate irrigation (D2) across all soil depths. In the 0–40 cm layer, RSA under D2 (190.33 cm^2^) was nearly threefold higher than that under D1 (50.27 cm^2^; *p* < 0.05) and substantially higher than that under D3. Similar patterns were observed in the 40–120 cm and 120–200 cm layers, where RSA under D2 exceeded that under the other treatments.

Root dry weight followed trends comparable to those of RSA. In the 0–40 cm layer, RDW under D2 (9.00 g) was nearly 29-fold higher than that under D1 (0.32 g) and markedly higher than that under D3 (1.44 g; *p* < 0.05). In deeper layers, RDW under D2 remained the highest among treatments, although absolute values decreased substantially with depth.

### 3.3. Isotopic Relationships Among Precipitation, Soil Water, and Xylem Water

**Figure 4:**
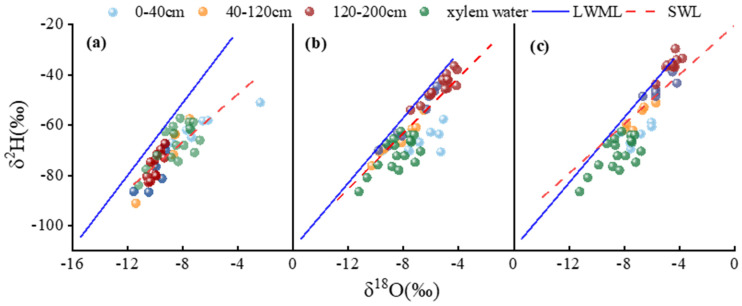
Dual-isotope comparison of precipitation, soil water, and citrus xylem water under different irrigation treatments. Panels (**a**), (**b**), and (**c**) represent shallow (D1), intermediate (D2), and deep (D3) treatments, respectively.

Comparative analysis of stable isotopes (δ^2^H–δ^18^O) revealed that the isotopic compositions of both soil water and xylem water deviated markedly from the local meteoric water line (LMWL: δ^2^H = 7.19 δ^18^O + 5.87; n = 26, R^2^ = 0.94, *p* < 0.01). This pattern indicates pronounced evaporative enrichment of soil water in citrus orchards located in red-soil hilly regions. The majority of soil water samples from all three treatments were distributed below the LMWL, suggesting that although precipitation represents the primary source of soil water, infiltrating rainfall underwent substantial evaporative fractionation throughout the growing season (Figure 4).

Marked differences were observed among treatments in the soil water evaporation lines (SWL), indicating that irrigation depth exerted a strong control on the intensity of soil water evaporation. Among the three irrigation treatments, D1 exhibited the lowest SWL slope (δ^2^H = 4.74 δ^18^O − 29.15; n = 30, R^2^ = 0.76, *p* < 0.01), reflecting the strongest evaporative fractionation and the most severe soil drying conditions. In contrast, D2 showed the steepest SWL (δ^2^H = 6.13 δ^18^O − 9.74; n = 30, R^2^ = 0.70, *p* < 0.01), suggesting that precipitation was directly replenished to the mid-soil layer (25–50 cm), thereby effectively alleviating evaporative fractionation in the surface soil. The SWL slope for D3 was intermediate (δ^2^H = 5.45 δ^18^O − 10.89; n = 30, R^2^ = 0.66, *p* < 0.01), indicating minimal evaporation of deep soil water per se, but moderate evaporative enrichment in shallow layers induced by upward capillary transport from deeper soil horizons. The rain-shelter film may have slightly altered the radiation and humidity conditions within the shelter, thereby exerting a minor influence on isotopic fractionation of surface soil water. However, soil layers below 40 cm were much less affected by evaporative processes. Moreover, the observed differences in isotopic fractionation among treatments (strongest in D1 and weakest in D2) were primarily governed by irrigation depth. Because all treatments were exposed to the same shelter conditions, the uniform disturbance introduced by the rain-shelter did not obscure the core treatment effects. Consequently, the water-source characteristics inferred from the isotopic analysis are considered reliable.

### 3.4. Seasonal Variation in Citrus Root Water Uptake Depth

**Figure 5:**
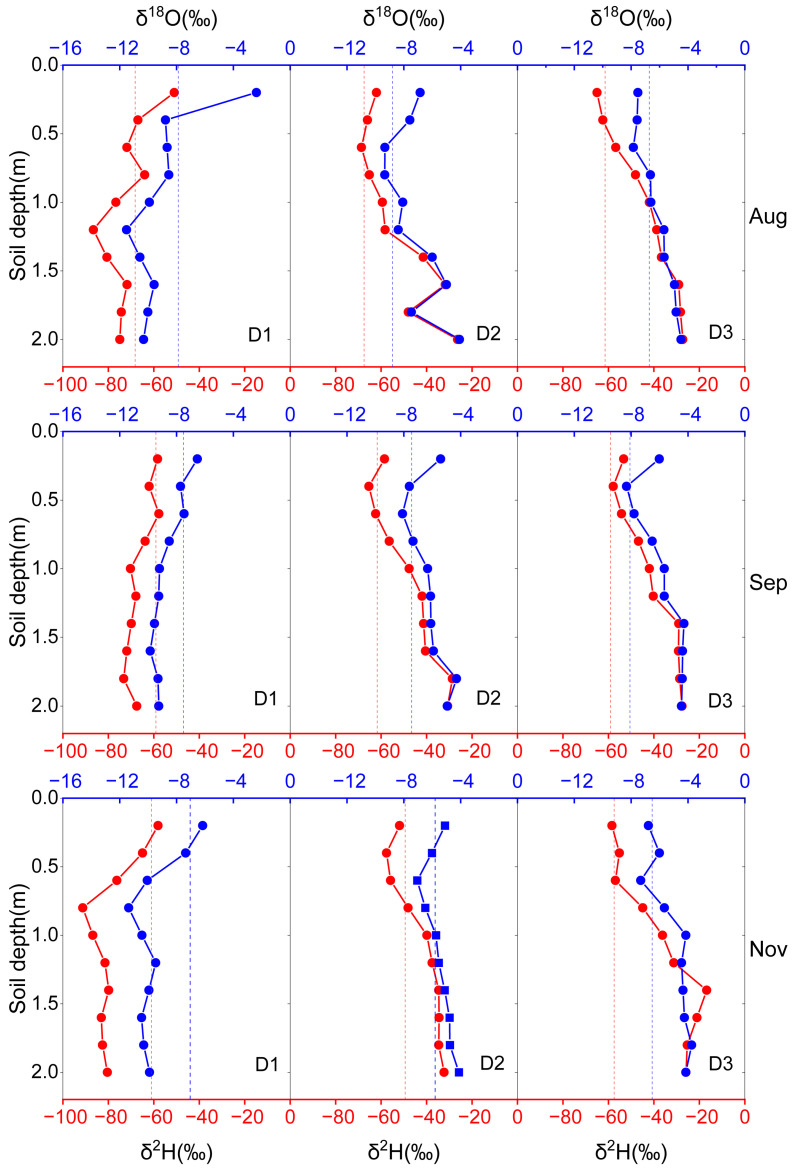
Relationships between citrus xylem and soil water isotopes under different treatments in August, September, and November (Red solid lines represent soil water δ^2^H, red dashed lines represent xylem water δ^2^H, blue solid lines represent soil water δ^18^O, and blue dashed lines represent xylem water δ^18^O).

The isotopic overlap between soil water and xylem water revealed a dynamic, month-to-month shift in the depth of citrus root water uptake (Figure 5). Under the shallow irrigation treatment (D1), root water uptake was primarily concentrated in the 40–90 cm soil layer in August. By September, the dominant uptake zone shifted upward to the 20–60 cm layer, and further shallowed to 20–40 cm by November. This progressive upward shift indicates a markedly increasing reliance of citrus trees on shallow soil water under D1. Under the intermediate irrigation treatment (D2), the depth of root water uptake exhibited pronounced seasonal variability. In August, water uptake was mainly derived from the 50–90 cm soil layer; by September, the dominant uptake zone migrated upward to 30–60 cm. In November, however, the uptake depth deepened substantially and expanded to encompass the 20–120 cm soil profile, indicating a transition from a relatively concentrated uptake zone to a vertically expanded water-uptake strategy. Similarly, under the deep irrigation treatment (D3), citrus root water uptake showed clear seasonal dynamics. In August, uptake was predominantly concentrated in the 40–100 cm soil layer. By September, the main uptake zone shifted upward to the 20–40 cm layer, and in November, root water uptake remained dominated by shallow soil water, primarily within the 20–50 cm layer.

### 3.5. Quantitative Analysis of the Contribution of Different Soil Layers to Citrus Root Water Uptake

**Figure 6:**
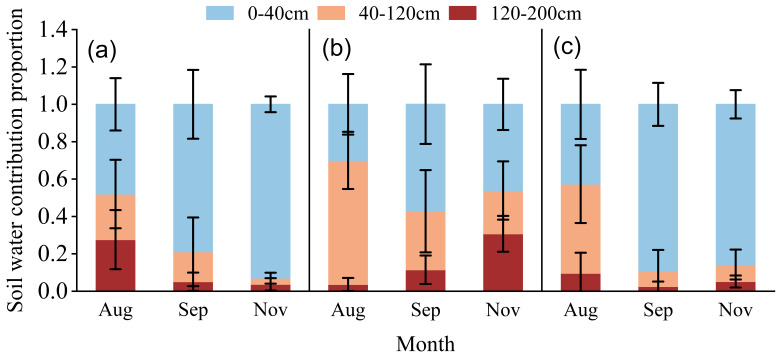
Contribution of different soil layers (0–40 cm, 40–120 cm, 120–200 cm) to citrus root water uptake under varying irrigation treatments. Panels (**a**), (**b**), and (**c**) represent shallow (D1), intermediate (D2), and deep (D3) irrigation treatments, respectively.

Based on the MixSIAR model outputs, citrus water-source utilization patterns differed markedly among irrigation depth treatments (D1–D3) (Figure 6). Under the shallow irrigation treatment (D1), citrus water uptake exhibited a pronounced reliance on shallow soil water, with its contribution increasing steadily from 48.00% in August to 93.00% in November, while the contribution of deep soil water declined significantly over the same period, from 27.60% to 3.70%.

In contrast, the intermediate irrigation treatment (D2) showed a more balanced and seasonally differentiated partitioning of water sources across soil layers. In August, shallow and mid-layer soil water contributed 30.00% and 66.40%, respectively; by September and November, the contributions of these two layers fluctuated synchronously and declined overall. Meanwhile, the contribution of deep soil water increased progressively from 3.60% to 30.70%, indicating that intermediate-depth irrigation enhanced the role of deep soil water in supporting citrus water uptake.

Under the deep irrigation treatment (D3), despite irrigation water being directly supplied to deeper soil layers, citrus water uptake was still dominated by shallow and mid-layer soil water. Their contributions ranged from 42.70% to 89.10% and from 47.70% to 8.40% between August and November, respectively, whereas the contribution of deep soil water remained consistently low (2.50–9.60%). This pattern suggests that deep irrigation did not substantially alter the preferential uptake of shallow to mid-layer soil water by citrus trees.

## 4. Discussion

### 4.1. Irrigation Depth Regulates Soil Water Persistence in the Active Root Zone

Soil water distribution in subtropical red-soil hilly orchards is jointly constrained by shallow effective soil depth, rapid drainage, and strong evaporative demand. Consistent with previous observations in red-soil and sloping orchard systems, soil water content generally increased with depth, while the surface layer remained highly vulnerable to drying [1,2,28]. However, our results further demonstrate that irrigation depth, rather than irrigation amount, exerts a dominant control on the persistence of soil water within the root zone.

In particular, the 40–120 cm layer emerged as the most responsive and hydraulically active zone, showing the strongest sensitivity to irrigation depth during the main growing season. This finding extends earlier studies that emphasized the role of mid-profile soil layers in buffering short-term surface drying in orchard and agricultural systems [5,8]. Compared with shallow irrigation, which primarily increased near-surface moisture but failed to sustain water availability due to rapid evaporation, and deep irrigation, which increased deep soil water storage with limited accessibility, intermediate irrigation delivered water directly to this active zone. As a result, it effectively balanced water accessibility and storage, leading to more stable root-zone moisture conditions.

These results highlight a fundamental trade-off between surface accessibility and deep storage that has often been overlooked in irrigation management studies focusing mainly on irrigation amount or frequency [3,4]. By explicitly isolating irrigation depth as a control factor, this study clarifies why moderate subsurface irrigation can outperform both shallow and deep irrigation in maintaining effective soil water availability in red-soil hilly orchards.

### 4.2. Root Architectural Responses to Depth-Specific Soil Water Availability

Root systems exhibited pronounced plasticity in response to irrigation depth, reflecting adaptive strategies to heterogeneous soil water environments. Similar to previous studies on citrus and other perennial fruit trees, root length density was largely concentrated in shallow soil layers across all treatments [13,29]. This pattern reflects the generally higher nutrient availability and oxygen supply in surface soils.

Beyond this common distribution pattern, our results reveal important differences in functional root traits. Intermediate irrigation consistently promoted greater root surface area and root biomass across the soil profile, indicating enhanced investment in absorptive capacity rather than simple elongation of roots. This finding aligns with the concept of root plasticity, whereby plants adjust root morphology and biomass allocation to optimize resource acquisition under varying water conditions [11,12]. In contrast, deep irrigation increased shallow root length density without proportionally enhancing root surface area or biomass, suggesting a strategy favoring short-term responsiveness to redistributed water rather than sustained uptake capacity.

Previous citrus studies have primarily quantified root distribution or root-zone extent [14,30], but have rarely linked root architectural traits to isotopically constrained water uptake sources. By combining root trait measurements with isotope-based uptake analysis, this study demonstrates that irrigation depth regulates not only where roots are located but also how effectively they function as water-absorbing interfaces across soil depths.

### 4.3. Evaporation-Driven Isotopic Separation and Its Implications for Uptake Depth

Dual stable isotope analysis revealed strong evaporative enrichment of soil water across all treatments, as indicated by systematic deviation from the local meteoric water line. Such patterns are widely reported in subtropical and semi-arid environments with high evaporative demand and limited soil water retention [16,31]. Importantly, irrigation depth substantially altered the slope of the soil water evaporation line, indicating differences in the intensity of non-equilibrium evaporation among treatments.

Shallow irrigation produced the lowest evaporation-line slope, reflecting strong surface evaporation and limited preservation of depth-dependent isotopic gradients. Similar isotopic responses under frequent surface wetting have been reported in irrigated agricultural soils, where rapid evaporation dominates soil water dynamics [32,33]. In contrast, intermediate irrigation increased the evaporation-line slope, suggesting that water supplied to the mid-soil layer bypassed the highly evaporative surface zone and reduced isotopic enrichment. Deep irrigation showed intermediate behavior, consistent with limited direct evaporation of deep soil water but partial upward redistribution and subsequent enrichment in shallow layers [34].

These results indicate that evaporation-driven isotopic separation among soil layers is a key mechanism linking irrigation depth to the vertical hierarchy of root water uptake. By preserving isotopic contrasts between shallow, intermediate, and deep soil water pools, intermediate irrigation enhances the potential for roots to access multiple water sources across depths.

### 4.4. Irrigation Depth Governs the Hierarchy and Plasticity of Root Water Uptake

Quantitative estimates from the MixSIAR model revealed pronounced differences in water-source utilization among irrigation treatments. Under shallow irrigation, citrus trees increasingly relied on shallow soil water as the season progressed, while deep soil water contributions declined markedly. This pattern is consistent with the “least-cost water use” principle, whereby plants preferentially exploit hydraulically accessible and energetically favorable water sources [35,36].

Intermediate irrigation promoted a more vertically integrated uptake strategy, with coordinated contributions from shallow, intermediate, and deep soil layers and an increasing role of deep soil water toward the late growing season. Similar adaptive uptake patterns have been reported for apple and olive orchards under regulated irrigation, where management practices enhanced root water uptake plasticity and drought resilience [23,37]. In contrast, deep irrigation failed to substantially increase deep-layer water uptake despite enhanced deep soil water storage, indicating that deep soil water primarily influenced uptake indirectly by modifying soil water potential gradients rather than serving as a dominant source.

This finding contrasts with reports from deep-rooted woody species and semi-arid ecosystems, where deep soil water or groundwater can contribute substantially to transpiration [38,39]. The discrepancy likely arises from differences in root system architecture, soil hydraulic connectivity, and management context. In red-soil hilly citrus orchards, shallow root dominance, rapid drainage, and limited upward hydraulic connectivity constrain the direct use of deep soil water. Consequently, simply increasing irrigation depth does not guarantee improved water uptake efficiency.

Overall, this study demonstrates that irrigation depth restructures the hierarchy and seasonal plasticity of root water uptake by jointly regulating soil water persistence, evaporative fractionation, and root functional traits. These mechanisms are expected to be transferable to other perennial orchard systems established on shallow, highly weathered soils under strong evaporative demand, providing a process-based basis for optimizing irrigation depth in water-limited agroecosystems.

## 5. Conclusions

This study systematically elucidated how irrigation depth regulates soil water distribution, root system traits, and root water uptake strategies in citrus orchards developed on subtropical red-soil hilly landscapes. Irrigation depth exerted a pronounced control on the spatiotemporal heterogeneity of soil water across the profile, with the 40–120 cm layer identified as the most sensitive and hydraulically active zone responding to irrigation inputs.

Compared with shallow and deep irrigation, intermediate irrigation at 50 cm consistently maintained higher and more stable soil water availability within the active root zone while substantially reducing surface-layer evaporative fractionation. Shallow irrigation enhanced short-term surface wetting but intensified non-productive evaporation and progressively constrained water uptake to shallow soil layers. In contrast, deep irrigation increased mid-to-deep soil water storage but contributed only marginally to direct root water uptake, indicating an indirect regulatory role rather than effective utilization by citrus roots.

Integration of stable isotope analysis and MixSIAR modelling revealed pronounced plasticity in citrus root water uptake strategies in response to irrigation depth. Intermediate irrigation promoted coordinated and seasonally adaptive water uptake from shallow, middle, and deep soil layers, whereas shallow irrigation led to an increasing reliance of roots on surface soil water, and increasing irrigation depth did not necessarily enhance water-use efficiency.

Overall, these findings demonstrate that irrigation depth exerts a stronger control over root water uptake patterns than irrigation amount by stabilizing water availability within the hydraulically active root zone and mitigating non-productive evaporative losses. For citrus orchards in red-soil hilly regions, optimizing subsurface irrigation depth—rather than increasing irrigation volume—represents an effective strategy to enhance water-use efficiency and improve the resilience of orchard water management under increasing climatic variability.

## Figures and Tables

**Table 1:** Soil hydraulic parameters of citrus orchard.

Soil Depth (cm)	Soil Bulk Density (g/cm^3^)	Organic Carbon (g/kg)	pH	Field Capacity θ_f_ (m^3^/m^3^)	Saturated Water Content θ_s_ (m^3^/m^3^)	Sand (%)	Silt (%)	Clay (%)
10	1.26	32.13	5.86	0.33	0.48	68.85	13.04	18.10
30	1.34	20.88	5.79	0.29	0.44	65.47	13.90	20.63
50	1.46	14.45	5.06	0.31	0.39	66.83	13.40	19.77
70	1.64	11.38	4.88	0.28	0.36	68.43	13.81	17.76
100	1.70	5.44	4.72	0.32	0.34	60.66	12.21	27.13

## Data Availability

The original contributions presented in this study are included in the article. Further inquiries can be directed to the corresponding authors.
